# Black Metals: Optical Absorbers

**DOI:** 10.3390/mi11030256

**Published:** 2020-02-28

**Authors:** Stefan Lundgaard, Soon Hock Ng, Yoshiaki Nishijima, Michael Mazilu, Saulius Juodkazis

**Affiliations:** 1 Optical Sciences Centre and ARC Training Centre in Surface Engineering for Advanced Materials (SEAM), School of Science, Swinburne University of Technology, Hawthorn, VIC 3122, Australia; soonhockng@swin.edu.au (S.H.N.); sjuodkazis@swin.edu.au (S.J.); 2 Melbourne Centre for Nanofabrication, the Victorian Node of the Australian National Fabrication Facility, 151 Wellington Rd., Clayton, 3168 VIC, Australia; 3 Department of Electrical and Computer Engineering, Graduate School of Engineering, Yokohama National University, 79-5 Tokiwadai, Hodogaya-ku, Yokohama 240-8501, Japan; nishijima@ynu.ac.jp; 4 Institute of Advanced Sciences, Yokohama National University, 79-5 Tokiwadai, Hodogaya-ku, Yokohama 240-8501, Japan; 5 SUPA, School of Physics and Astronomy, University of St. Andrews, St. Andrews KY16 9SS, UK; 6 Tokyo Tech World Research Hub Initiative (WRHI), School of Materials and Chemical Technology, Tokyo Institute of Technology, 2-12-1, Ookayama, Meguro-ku, Tokyo 152-8550, Japan

**Keywords:** metamaterial, optical absorber, thin-film optics

## Abstract

We demonstrate a concept and fabrication of lithography-free layered metal-SiO_2_ thin-film structures which have reduced reflectivity (black appearance), to as low as 0.9%, with 4.9% broadband reflectance (8.9% for soda lime) in the 500–1400 nm range. The multi-layered (four layers) thin-film metamaterial is designed so that optical impedance matching produces minimal reflectance and transmittance within the visible and infra-red (IR) spectral region for a range of incident angles. The structure has enhanced absorbance and is easily tuned for reduced minimal transmission and reflection. This approach should allow for novel anti-reflection surfaces by impedance matching to be realized.

## 1. Introduction

Photonic metamaterials are providing new capabilities for highly efficient and tunable solutions in photonic devices with light phase delay and intensity modulation for resonators, waveguides, absorbers, switches, and detectors [1,2,3]. Perfect absorbers are of interest within the metamaterial family due to wavelength specific applications such as solar cells, photo-detectors, optical filters, and coatings. Meta-materials/surfaces have sub-wavelength scale films/structures and can create unusual optical properties, e.g., left-handed (negative refractive index) materials [4]. By tailoring real and imaginary parts of the refractive index, a new field of topological photonics has emerged where breaking optical reciprocity and parity-time symmetry is achieved [5,6]. Recently, modeling and fabrication of metasurfaces with designed amplitude and phase delay patterns can be performed using open access software which outputs design files required for popular electron-beam or projection lithography techniques [7]. These lithography based fabrication methods for nano-meter scale features are slow, costly, and require high fidelity.

Devices and structures that modulate and manipulate waves can be made for sound/vibrations, light, and electrical waves [8,9,10]. In particular, impedance matching is a method of preventing wave reflection, a technique used heavily in electronics. Metamaterials and thin-film technology has made it possible for the impedance of fabricated devices to match the impedance of bands in the electromagnetic spectrum [11,12]. Negative refractive index materials in the optical spectrum have previously been realized through complex patterns via lithography and thin-film deposition within the optical spectrum [13,14]. Now, a novel approach for simple minimal-layer films to realize impedance matching in the optical domain of the electromagnetic spectrum has been realized.

Here, we use a lithography-free multi-layer approach to fabricate perfect absorbers by an impedance matching principle for light in the visible range [11,15]. The streamlined and simple fabrication process reduces time and cost over standard lithography-based approaches. Thin film techniques allow for these metamaterials to be designed at sub-wavelength thicknesses, creating highly optimized structures, tuned for a particular spectral range. By changing nano-film material and thickness, colors are produced and particular wavelength bands are selectively reflected. Appearance of color is a consequence of the optical impedance mismatching at different wavelengths. These optical absorbers, using only metal and dielectric materials, appear black and thus are coined ’black metals’. By realizing the impedance of the incident wave and matching the respective impedance of the multi-layer metamaterial, a reduced reflectance is determined, opening up a new method for anti-reflection coatings, solar-cells, optical absorbing structures, and photo-thermal applications [16,17].

## 2. Theory

**Figure 1:**
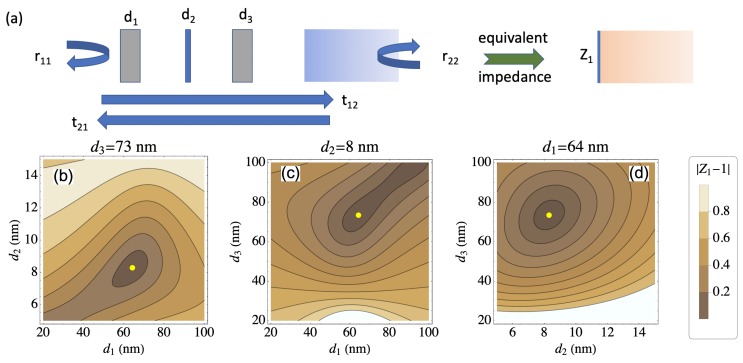
(**a**) Schematic representation of the structure consisting of three layers (silica, metal, silica) with respective thicknesses ($d_{1}$, $d_{2}$ and $d_{3}$) on top of the thick metal layer. The field coefficients are defined in the text. (**b**–**d**) impedance mismatch $|Z_{1}-1|$ as a function of the different layer thicknesses for normal incidence at a wavelength (λ=500 nm). The yellow dot corresponds to the layer thicknesses ($d_{1}=64$ nm, $d_{2}=8$ nm, $d_{3}=73$ nm) minimizing overall back-reflection $r_{11}$.

**Figure 2:**
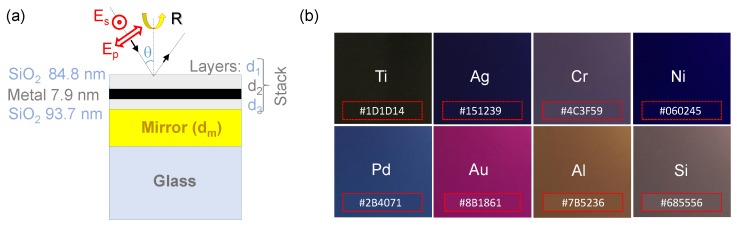
Black metals. (**a**) schematic of the structure of stacked nano-films [11] investigated for reflectance and absorbance control; the numerically optimized thicknesses for black-Ti are shown (see Section 4.1 for details); (**b**) photos of reflected colors under fluorescent white light illumination for various materials in layer two ($d_{2}$). Hexadecimal color codes with the respective colors are shown on the images. The samples are all fabricated with the same target thicknesses for all layers: layer-1 $d_{1}=84.8$ nm, layer-2 $d_{2}=7.9$ nm, layer-3 $d_{3}=93.7$ nm. The thickness of the back Ti mirror was $d_{m}=100$ nm.

The low reflectance metamaterials have been designed as optical absorbers, consisting of a simple stacked layer of dielectric-metal-dielectric on a metal mirror (Figure 1 and Figure 2a). Layer thicknesses are optimized through the help of simulations in Matlab (MathWorks, Natick, MA USA) which determines the optical impedance of the sub-wavelength thin-films. The optical absorbers are then fabricated via standard thin film deposition techniques. Using different materials for $d_{2}$ and thicknesses for layers $d_{1–3}$ creates a change in impedance of the absorber and hence a color change (Figure 2b).

A scattering matrix is used in conjunction with material permittivity and permeability properties to determine the effective zero reflectance R=0. This zero reflection regime is realized when the layers create an effective impedance that matches that of the incident optical wave in air (or other medium) on the incident side. This interface impedance corresponds to the image impedance defined in reference [11] as:

(1) $$\begin{array}{ccc} Z_{1} & = & \sqrt{\frac{\left(r_{22}r_{11}-r_{11}-r_{22}-t_{12}t_{21}+1)(r_{22}r_{11}+r_{11}-r_{22}-t_{12}t_{21}-1\right)}{\left(r_{22}r_{11}-r_{11}+r_{22}-t_{12}t_{21}-1)(r_{22}r_{11}+r_{11}+r_{22}-t_{12}t_{21}+1\right)}}. \end{array}$$

where the field transmission ($t_{12}$, $t_{21}$), and reflection ($r_{11}$, $r_{22}$) coefficients (represented in Figure 1) are calculated to take into account the whole structure from the first air–silica (silicon dioxide, SiO_2_) interface to the final silica–metal interface.

The layer thicknesses are sub-wavelength which allows for an incident wave to perceive the material as having a different impedance than that of the individual materials used. Calculations were performed for low reflectivity and with a larger angular range down to the Brewster angle using the method described in reference. [11]. The refractive index of the materials were taken from the public database [18] and the popular finite difference time domain (FDTD) program Lumerical (Lumerical Inc., Vancouver, BC, Canada) as indicated where applicable. Electric field enhancement simulations using Lumerical is also performed to investigate location and enhancement factor induced by incident light for surface enhanced Raman spectroscopy (SERS).

Reflection spectra simulations are calculated by stepped iterations across the visible spectrum and over a range of layer thicknesses. The wavelength is stepped in intervals of 1 nm from 300–1000 nm, and the layer thicknesses ($d_{1–3}$) are in stepped intervals of 1 nm for $d_{1}$, 0.5 nm for $d_{2}$, and 1 nm for $d_{3}$. Spectral plots and surface contour maps for visualization are realized and material layer thicknesses are then extrapolated for the zero reflectance R=0.

## 3. Experimental

### 3.1. Fabrication

Soda lime substrates of 1 mm thickness with deposited thin films of metal and SiO_2_ layers were fabricated to produce the optical absorbers. The films were deposited using an *AXXIS* physical vapor deposition (PVD) chamber (Kurt J. Lesker Company, Jefferson Hills, PA, USA) via electron beam evaporation (EBE) deposition with 4N (99.99%) material purity minimum and a base pressure of at least $5\times 10^{-6}$ Torr. Film thickness was determined by quartz crystal monitors (QCM) and tooling factors were calibrated by measuring step height of the thin films using atomic force microscopy (AFM). The simulated and initial optical absorber was fabricated using titanium (Ti) and SiO_2_. Other materials such as silver (Ag), chromium (Cr), nickel (Ni), palladium (Pd), gold (Au), aluminium (Al), silicon (Si), and titanium dioxide (titania, TiO_2_) in place of Ti for layer $d_{2}$ were tested for production of colored films as shown in Figure 2b. The hexadecimal values are extrapolated from color averages in the images when taken at 10° from normal incidence in white fluorescent lighting. The thicknesses shown correspond to the calibrated set point of the evaporation conditions.

### 3.2. Characterization

Reflection *R* and transmission *T* spectra of the absorber stacks were measured using a PerkinElmer 70 *Lambda 1050 WB* (Waltham, MA, USA) UV-Vis-IR spectrometer. The UV spectrum is generated by a D_2_ lamp (until 319.2 nm) and then a tungsten lamp is used in the Vis-IR range until a 1400 nm wavelength. The UV-Vis region is detected with a photo-multiplier tube until 870 nm and then longer wavelengths are detected using an InGaAs sensor. All spectra are normalized against a 100% Transmission, 0% Absorbance scan inside a 150 mm integration sphere using a Labsphere Spectralon ®EPV diffuse reflectance standard (North Sutton, NH, USA).

## 4. Results

### 4.1. Modeling

Multi-layer stacks of two alternating dielectrics—one-dimensional, photonic crystal—can achieve high reflectivity R→1 (T→0) and, ideally, without absorption losses A=0. However, fabrication of such stack mirrors, usually used as spectrally narrow filters, is a formidable technical challenge due to larger number of layers N∼100. The larger the number of layers used, the greater the reflectivity that can be achieved. For the opposite task, which is to obtain minimum reflectance *R*, multi-layer absorbers can be used.

For example, a 30-layer absorber with a 60.4 nm SiO_2_ top layer with alternating layers of 10 nm Ti and decreasing thickness of SiO_2_ starting from 119.6 nm down to 50 nm in steps of 2.4 nm can effectively reach R=1% [11] (not shown here). The total thickness of such a 30-layer Ti/SiO_2_ stack is ∼2.9 μm.

**Figure 3:**
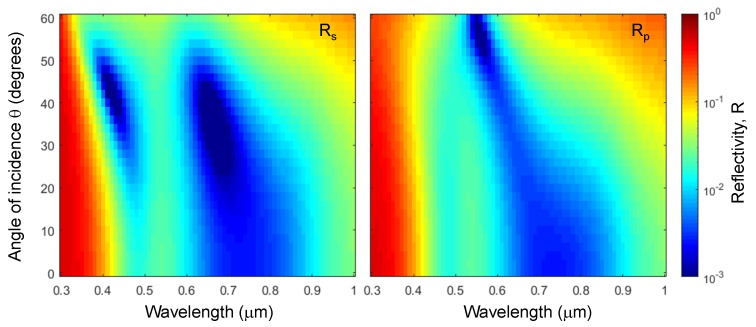
Plots of the simulated of reflectivity for s- and p-polarisations $R_{s},R_{p}$ at different angles of incidence 0–60 θ within the 0.3–1 μm spectral window. The stack was SiO_2_/Ti/SiO_2_ with thicknesses of $d_{1}=84.8$ nm, $d_{2}=7.9$ nm, $d_{3}=93.7$ nm on a 100-nm-thick Ti mirror on a soda lime glass substrate (see stack geometry in Figure 2a).

In this study, further numerical optimization was performed to reduce the total thickness whilst retaining optical impedance matching within the UV-IR spectral range. Subsequent optimizations found that a three layer stack of alternating SiO_2_-Ti with thicknesses of 84.8 nm–7.9 nm–93.7 nm ($d_{1}$–$d_{2}$–$d_{3}$) on a 100-nm-thick mirror produce a similar result with a total thickness of only 386.4 nm (Figure 2a). An angular dependence is also shown in Figure 3 for both s- and p-polarizations. There is an effective reduced reflectance from normal incidence which tapers off when approaching ∼60° from normal incidence. While fabrication should have high process control, optimal structure thicknesses should allow for flexibility of angular variation when used in relevant applications.

Each of the three layers affect the reflectance *R* and transmittance *T* spectra. The top three layers determine the impedance of the total system, defining which wavelengths are not reflected. The mirror layer and substrate absorb most of the non-reflected incident light.

**Figure 4:**
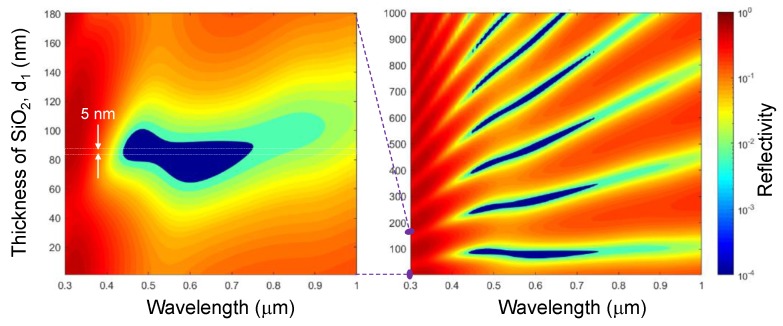
Plots of the simulated reflectivity *R* spectrum of a thin-film stack (Figure 2a) SiO_2_/Ti/SiO_2_ optical absorber, where the mirror layer was Ti *d_m_* = 100 nm; note the logarithmic scale of *R*. The thickness of the first layer SiO_2_ $d_{1}$ was varied from 0 to 180 nm (left panel) to determine the minimum of *R* which was found at $d_{1}=84.8$ nm. The darkest blue region has ≤ 1.3% reflectivity in the 430–960 nm spectral range. Other parameters are: $d_{2}=7.9$ nm, $d_{3}=93.7$ nm, $d_{m}=100$ nm and normal incidence θ=0°.

**Figure A1:**
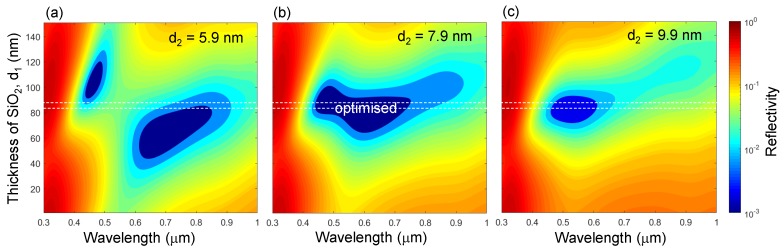
Simulations of the $d_{1}$ (y-axis) SiO_2_ effect on the performance of SiO_2_/Ti/SiO_2_ layers where layer-2 (Ti) was $d_{2}=5.9$ nm (**a**), $d_{2}=7.9$ nm (**b**) and $d_{2}=9.9$ nm (**c**) thick (structure is shown in Figure 2a); thickness $d_{3}=93.7$ nm. Normal incidence θ=0°.

**Figure A2:**
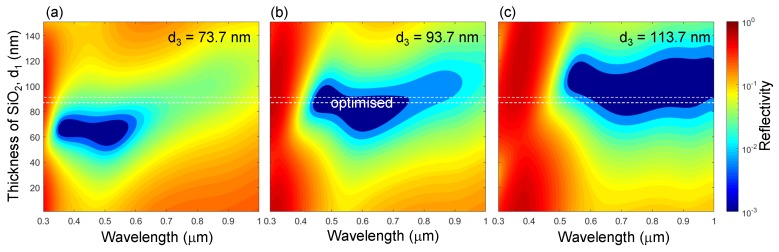
Simulations of the $d_{1}$ (y-axis) and *d*_3_ SiO_2_ thickness effect on the performance of SiO_2_/Ti/SiO_2_ layers. Layer-3 (SiO_2_) is varied in steps of 20 nm: $d_{3}=73.7$ nm (**a**), $d_{3}=93.7$ nm (**b**) and $d_{3}=113.7$ nm (**c**); structure is shown in Figure 2a; thickness $d_{2}=7.9$ nm. Normal incidence θ=0°.

**Figure A3:**
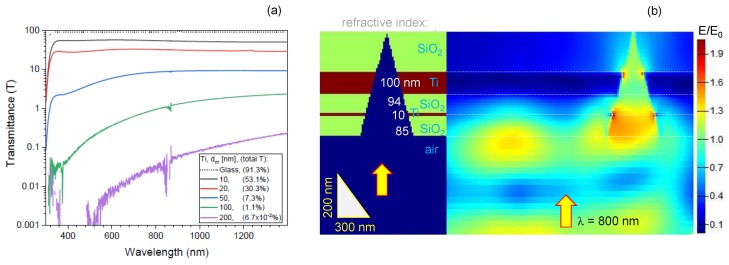
(**a**) Titanium mirror layer ($d_{m}$) thickness effect on the transmittance of incident light. Total integral transmittance *T* [%] is calculated from area under the curve; (**b**) finite difference time domain (FDTD, Lumerical) simulation on light localization in the optimized black-metal stack using Ti. The triangular groove is inserted for visualization of possible changes to light intensity in a simulated laser ablated region. In the pristine region of the impedance matched section, light is localized on the front interface (light propagation is shown by arrow). E-field on incident plane wave $E_{0}=1$ V/m.

The effect of the first layer ($d_{1}$) thickness variation is shown in Figure 4, where increasing Fresnel reflections causes oscillations in the spectra. Therefore, the layer thickness can only be within a small range to have broadband zero reflectance (R→0). The thickness of layers two ($d_{2}$) and three ($d_{3}$) affects the optimal conditions for zero reflectance in different ways, but all layers affect the impedance of the absorber. As shown in Figure A2 by decreasing layer $d_{3}$ thickness, the low reflectance region blue-shifts and similarly by increasing the thickness it will red-shift. Layer $d_{2}$ thickness is material dependent and can remain the same thickness within the visible spectrum. The overall minimum reflectance of R≈1% when $d_{2}$ and $d_{3}$ varied only slightly, significant changes are evident with a 20% thickness variation (see Appendix A section for details: Figure A1 and Figure A2). The mirror layer ($d_{m}$), by itself, is a highly lossy layer where the majority of extinction occurs; the $d_{m}=100$ nm thick Ti layer on glass has a transmission of T<1.1% from 200–1400 nm (Figure A3. The impedance of the incident light is wavelength dependent, thus each of the top three layers of the absorber need to vary in thickness for optimized low reflectivity performance across the spectral range considered. This is a readily achievable task and practical realization of designed layers is shown next.

### 4.2. Fabrication of Black-Metal Absorbers

The optimized thicknesses for layers $d_{1,2,3}$ to achieve R→0 performance (A→1 perfect absorber) is outlined in Section 4.1. These layer thicknesses were established by measuring step height for calibration of patterned metal and dielectric films deposited on silicon or soda lime glass. This was determined outside of a clean room environment by semi-contact tapping-mode atomic force microscopy (AFM) with a 10 nm radius silicon tip. The Ti and SiO_2_ films were measured to have an average surface roughness (Sa) of 0.07 nm and 1.7 nm (95% confidence range) on silicon with peak to peak (Sy) of 0.5 nm and 20 nm, respectively. The glass substrates had a measured Sa of 4.2 nm (95% confidence range) and Sy of 62 nm. Since different metals have different surface energies, deposition of thin ∼1 nm films is always sensitive to substrate material and its surface texture down to the nanoscale. We used e-beam evaporation and considerably high vacuum $5\times 10^{-6}$ Torr which facilitates formation of smooth layers of high purity and small grain size. The surface roughness of titanium metal used in this study deposited on silicon for thickness calibration was Δd=±0.25 nm ($\frac{1}{2}Sy$) over the length of ∼5 μm. Thickness of the deposited films is given by the QCM method from the linear fit mass vs. time at the chosen deposition rate (set conditions). Precision of such method was ±0.1 nm where the most significant process control is from tooling of the QCM. The second process control taken into account is a read delay of the QCM. Where the shutter closes once, the thickness set point is reached, but, due to continuous read-out, there is a measurement delay (and read error as well due to temperature variation of the QCM during deposition) of the actual thickness. This thickness overshoot (read-out delay) was found to have a linear relationship with deposition rate specific to each material. Accommodating this overshoot, the precision of film deposition thickness read out by the crystal monitor was Δd=0.1 nm (defined as a mass of the deposited film) over multiple deposition runs. Therefore, the total thickness margin of error across multiple deposition runs is Δd=0.35 nm.

**Figure 5:**
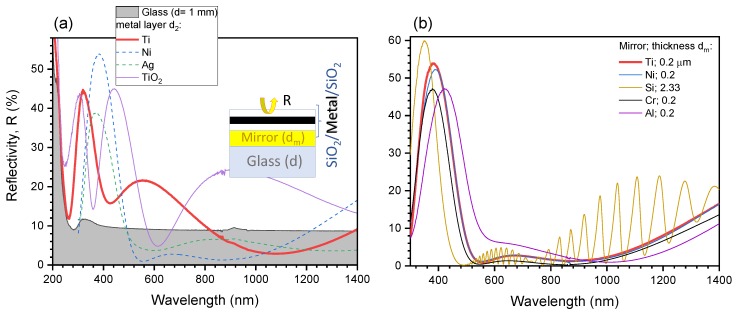
(**a**) Reflection spectra for various $d_{2}$ materials when $d_{2}=7.9$ nm. A soda lime substrate reference spectra in shown in background; (**b**) reflection spectra for a Ni $d_{2}$ layer with various metals substituted in the mirror layer of $d_{m}=200$ nm; for silicon (from a silicon on insulator substrate), $d_{m}\approx 2.33\;\mu$m. The thickness evaluated from two interference peaks $\lambda_{1}=974.74$ nm and $\lambda_{2}=1037.11$ nm is $d_{m}=\frac{\lambda_{1}\lambda_{2}}{2n(\lambda_{2}-\lambda_{1})}\approx 2.21\;\mu$m calculated for the refractive index n=3.673 [19].

The designed Ti nano-layer structure (Figure 2a) was fabricated and the reflectance spectrum (Figure 5a) was found to sharply decrease at wavelengths longer than 400 nm, matching the simulated spectrum in Figure 4. The actual thickness $d_{2}$ (Ti) was most probably less than the simulated by ∼1 nm, which can explain the higher reflectivity and saddle shape around 600 nm wavelength as shown in Figure A1 simulations. Due to the inaccurate thicknesses, the reflectance *R* only becomes smaller than that of the glass (<0.1) only after 821 nm.

In comparison, a low reflectance for the Ag and Ni layers over the 500–1200 nm spectral window was observed in the fabricated three-layer films on a Ti-mirror (Figure 5a). The integral (area under the curve) reflectance of the soda-lime glass in the 500–1400 nm range is 8.9%. The Ti $d_{2}$ layer sample has a reduced reflectance in the 821–1385 nm range but is greater below 821 nm and has an overall reflectance of 9.3% in the 500–1400 nm range, thus, the layer thicknesses require further optimization to match simulations. Similarly, for the Ag $d_{2}$ and the Ni $d_{2}$ samples, reflectance in the 500–1400 nm range are 4.9% (minimum 3.6% @ 1307 nm) and 4.9% (minimum 0.9% @ 556 nm) respectively, reducing reflectance in comparison down to 55.1% and 54.9% of the soda-lime, respectively.

It is noteworthy that the structure was not simulated nor optimized for Ag and Ni and was deposited for comparison but not as calculated impedance matched structures. Figure 2b shows appearance of the black-metal mirrors whose reflectance is presented in Figure 5a. Titania (TiO_2_) was also tested as layer-2 since Ti can be oxidized after exposure to room conditions and the SiO_2_ layers. Strong reflectivity around 400 nm (3.1 eV) was observed. This is consistent with band-gap edge absorption of titania. At the strong absorption, low reflectivity R=1−A−T is expected when T→0 as in the studied case. At the condition of R→0, light localization on the front surface was revealed by numerical simulations with intensity enhancement $E^{2}\approx 4$ (Figure A3b). This can be a useful feature for Raman scattering since front-irradiation causes the intensity maximum ∼λ/4 before the interface which is not optimum for sensors, and the back-side (through the substrate) has to be used for the exact intensity maximum positioned on the interface [20].

The influence of the mirror-layer (originally 100 nm of Ti) with a SiO_2_/Ni/SiO_2_ stack was tested by evaporating films of different materials (Figure 5b), where the optimized layer structure is shown in Figure 2a. Nickel was chosen as layer $d_{2}$ due to the lowest broadband reflectance performance, shown in Figure 5. The reflectivity for all mirror materials tested (excluding Si) remains lower than that of soda lime glass within ∼500–1200 nm. Different metals behaved similarly with the strongest difference at the UV spectral range where free-electron density differences become important. Interestingly, a 2.3 μm Si mirror layer showed similar performance up to 900 nm wavelengths (close to the absorption band-gap of 1.12 eV). The reflectance obtained with the mirror layers $d_{m}$ = 200 nm of Ni, Cr, and Ti all have similar profiles, where Al was slightly red-shifted perhaps due to interface oxidation.

## 5. Discussion

It is usual to expect that metal coatings will act as reflective mirrors. Here, we show that nano-engineered silica-metal-silica layers with a controlled thickness deposited on a mirror renders its surface black (dark) and delivers a low reflectance R→0 regime.

Simulations and experimental spectra have variation differences quantitatively but agree well qualitatively for the expected reflectivity spectral line shape. Exact layer thicknesses are yet to be achieved as detailed in Figure 5a as the minimum reflectance achieved is with a nickel $d_{2}$ layer. Fabrication error is on the order of ±0.35 nm due to thin film grain formation and lateral uniformity of the film even when the QCM tooling accuracy defining mass is ±0.1 nm as tested across multiple deposition runs. Step height measurements using AFM are accurate; however, a change in interface profile when a layer is deposited over the top is not well known.

Material of the mirror-layer had a minimal effect on the reflection spectra, and oxidation of the mirror at the interface with the SiO_2_ will occur for the reactive metals. Oxidation of a thin nanometer-scale Ti layer can have influence on the optical constants (n+iκ). Moreover, we have demonstrated that, when thickness of deposited films is ∼10 nm, the nanoscale roughness has a strong influence and optical performance of metamaterial absorbers at the IR spectral window departs from numerical predictions based on optical constants [21]. By invoking surface roughness at the interface and top surface, it was possible to obtain more closely matching transmission spectra [21]. Future work is necessary to reach more quantitative match between theory and experiment and will be carried out in the nearest future.

A possible application of color change as a result of optical impedance mismatch in black metal layers can be in use for visualization of hydrogen uptake in sensors where Pd and Pd-containing alloys take up H^2+^ and H^+^ with strong lattice change of the host metal [22]. Figure A3b shows numerical simulations for cross sections where Ti metal layers are exposed to air (ambience) by triangular incisions which simulate laser ablated trenches. Light field enhancement and depolarization at the metal–air interface facilitates electron charge transport through the interface required for molecular H_2_ to enter metal phase in the ionic forms H^2+^ and H^+^ [22].

## 6. Conclusions and Outlook

Reflectivity of the “black-metals” are measured to be lower than that of glass over the spectral range of 500–1000 nm which can be made by a simple silica-metal-silica sandwich deposited over a back-reflecting mirror. Glass was measured to have a broadband reflectance of ∼8.9% (>400 nm) and the impedance matched layers are able to reach a reduced reflectance in comparison. The broadband reflectance from 500–1400 nm values measured for $d_{2}$ layer materials are Ti: 9.3%, Ag: 4.9%, and Ni: 4.9%. The mirror material was not the most critical parameter for the spectral bandwidth. The thickness of a thin metal film of the second layer $d_{2}\approx 8$ nm is the most sensitive parameter defining the reflection spectrum. Considerable departure of spectral response of the fabricated structure from that simulated is attributed to imperfections at the interface regions as previously observed for IR metamaterial absorbers [21]. Further optimizations of film thickness accuracy will produce optimal results for a zero reflectance (R→0) optical metamaterial.

Fabrication of thin films with a specific optical function and making them chemically releasable for opto-mechanical manipulation [23] could also be used for metamaterial coatings studied in this work. How the low emissivity of metals (0.02–0.05) as compared to that of black-body (emissivity of 1) is changed for the antireflective black-metal surfaces is the next intriguing quest. Following the experimentally demonstrated Kirchhoff’s law for IR metamaterial absorbers, when a strong absorbance at a spectrally narrow IR band showed the proportional emittance E≈A [24], the expectation is that silica–metal–silica structures on a mirror would be a broad band thermal emitter. 

## Appendix A. Parameter Study of: 1) *d*_1_ Dependence on *d*_2,3_ and 2) *d_m_*

Effect of the second layer ($d_{2})$, Ti thickness variation shown in Figure A1. The layer thickness was varied by 2 nm (∼20% of the layer thickness) and the third layer ($d_{3}$), SiO_2_ impact heavily the impedance of the system and define where the zero reflectance region occurs along the optical spectrum. By increasing $d_{3}$ (SiO_2_) thickness, the zero reflection region will red-shift and a greater fraction of the IR spectrum will have reduced reflectance, but more of the UV-Vis spectrum will be reflected. The reverse is true when the $d_{3}$ thickness is decreased, blue shifting the zero reflectance region as shown in Figure A2.

Just with the layers $d_{3}$ and $d_{m}$ (no impedance matching), Figure A3 shows the transmission spectra at normal incidence for increasing Ti mirror layer thicknesses. Total area under the curve integral relative to 100% transmission is calculated to give a transmittance of 53% for $d_{m}$ = 10 nm, 1.1% for $d_{m}$ = 100 nm, and 6.7 × 10^−2^% for $d_{m}$ = 200 nm. If, for example, the d=100 nm thickness was changed to Au, then the transmission should be calculated as $T=e^{-\alpha d}\equiv e^{-(4\pi \kappa /\lambda )d}=0.11\%$; here, the absorption coefficient α is expressed via the extinction coefficient κ (the complex refractive coefficient (n+iκ)), which has a value of κ=3.4332 for wavelength λ=633 nm [25]. Control of the transmittance by varying the $d_{m}$ layer thickness is novel and provides an exponential broadband reduction across the visible spectrum.

## References

[1] Yu N., Capasso F. (2014). Flat Optics with Designer Metasurfaces. Nat. Mater..

[2] Kats M.A., Capasso F. (2016). Optical absorbers based on strong interference in ultra-thin films. Laser Photonics Rev..

[3] Holloway C.L., Kuester E.F., Gordon J.A., O’Hara J., Booth J., Smith D.R. (2012). An Overview of the Theory and Applications of Metasurfaces: The Two-Dimensional Equivalents of Metamaterials. IEEE Antennas Propag. Mag..

[4] Zheludev N.I., Kivshar Y.S. (2012). From metamaterials to metadevices. Nat. Mater..

[5] El-Ganainy R., Makris K.G., Christodoulides D.N., Musslimani Z.H. (2007). Theory of Coupled Optical PT-Symmetric Structures. Opt. Lett..

[6] Özdemir K., Rotter S., Nori F., Yang L. (2019). Parity–Time Symmetry and Exceptional Points in Photonics. Nat. Mater..

[7] Dharmavarapu R., Ng S.H., Eftekhari F., Juodkazis S., Bhattacharya S. (2020). MetaOptics: Opensource software for designing metasurface optical element GDSII layouts. Opt. Express.

[8] Beranek L.L., Sleeper H.P. (1946). The Design and Construction of Anechoic Sound Chambers. J. Acoust. Soc. Am..

[9] Liu N., Mesch M., Weiss T., Hentschel M., Giessen H. (2010). Infrared perfect absorber and its application as plasmonic sensor. Nano Lett..

[10] Matthaei G.L., Young L., Jones E. (1964). Microwave Filters, Impedance Inverters and Coupling Structures.

[11] Mazilu M., Dholakia K. (2006). Optical impedance of metallic nano-structures. Opt. Express.

[12] Noh H., Chong Y., Stone A.D., Cao H. (2012). Perfect coupling of light to surface plasmons by coherent absorption. Phys. Rev. Lett..

[13] Valentine J., Zhang S., Zentgraf T., Ulin-Avila E., Genov D.A., Bartal G., Zhang X. (2008). Three-dimensional optical metamaterial with a negative refractive index. Nature.

[14] Stomeo T., Casolino A., Guido F., Qualtieri A., Scalora M., Orazio A.D., Vittorio M.D., Grande M. (2019). 2D Dielectric Nanoimprinted PMMA Pillars on Metallo-Dielectric Films. Appl. Sci..

[15] Lawrence F.J., Botten L.C., Dossou K.B., Martijn de Sterke C. (2008). Antireflection coatings for two-dimensional photonic crystals using a rigorous impedance definition. Appl. Phys. Lett..

[16] Spinelli P., Hebbink M., Waele R.D., Black L., Lenzmann F., Polman A. (2011). Optical Impedance Matching Using Coupled Plasmonic Nanoparticle Arrays. Nano Lett..

[17] Kim K.H., Park Q.H. (2013). Perfect anti-reflection from first principles. Sci. Rep..

[18] Refractive Index Database. https://refractiveindex.info/.

[19] Aspnes D., Studna A.A. (1983). Dielectric functions and optical parameters of Si, Ge, GaP, GaAs, GaSb, InP, InAs and InSb from 1.5 to 6.0 eV. Phys. Rev. B.

[20] Jayawardhana S., Rosa L., Juodkazis S., Stoddart P.R. (2013). Additional Enhancement of Electric Field in Surface-Enhanced Raman Scattering due to Fresnel Mechanism. Sci. Rep..

[21] Nishijima Y., Balčytis A., Naganuma S., Seniutinas G., Juodkazis S. (2018). Tailoring Metal and Insulator Contributions in Plasmonic Perfect Absorber Metasurfaces. ACS Appl. Nano Mater..

[22] Nishijima Y., Shimizu S., Kurihara K., Hashimoto Y., Takahashi H., Balcytis A., Seniutinas G., Okazaki S., Juodkazytė J., Iwasa T. (2017). Optical readout of hydrogen storage in films of Au and Pd. Opt. Express.

[23] Grineviciute L., Tolenis T., Ryu M., Moein T., Ng S.H., Katkus T., Maksimovic J., Drazdys R., Morikawa J., Juodkazis S. (2019). Releasable Micro-Waveplates. arXiv Preprint.

[24] Nishijima Y., Balčytis A., Naganuma S., Seniutinas G., Juodkazis S. (2019). Kirchhoff’s Metasurfaces Towards Efficient Photo-Thermal Energy Conversion. Sci. Rep..

[25] Johnson P.B., Christy R.W. (1972). Optical Constant of the Nobel Metals. Phys. Rev. B.

